# From Prototype to Inference: A Pipeline to Apply Deep Learning in Sorghum Panicle Detection

**DOI:** 10.34133/plantphenomics.0017

**Published:** 2023-01-16

**Authors:** Chrisbin James, Yanyang Gu, Andries Potgieter, Etienne David, Simon Madec, Wei Guo, Frédéric Baret, Anders Eriksson, Scott Chapman

**Affiliations:** ^1^ School of Agriculture and Food Sciences, The University of Queensland, Brisbane, Australia.; ^2^ School of Information Technology and Electrical Engineering, The University of Queensland, Brisbane, Australia.; ^3^ Queensland Alliance for Agriculture and Food Innovation, The University of Queensland, Brisbane, Australia.; ^4^ Arvalis, Institut du Végétal, Paris, France.; ^5^ Graduate School of Agricultural and Life Sciences, The University of Tokyo, Tokyo, Japan.; ^6^ Institut National de la Recherche Agronomique, Paris, France.

## Abstract

Head (panicle) density is a major component in understanding crop yield, especially in crops that produce variable numbers of tillers such as sorghum and wheat. Use of panicle density both in plant breeding and in the agronomy scouting of commercial crops typically relies on manual counts observation, which is an inefficient and tedious process. Because of the easy availability of red–green–blue images, machine learning approaches have been applied to replacing manual counting. However, much of this research focuses on detection per se in limited testing conditions and does not provide a general protocol to utilize deep-learning-based counting. In this paper, we provide a comprehensive pipeline from data collection to model deployment in deep-learning-assisted panicle yield estimation for sorghum. This pipeline provides a basis from data collection and model training, to model validation and model deployment in commercial fields. Accurate model training is the foundation of the pipeline. However, in natural environments, the deployment dataset is frequently different from the training data (domain shift) causing the model to fail, so a robust model is essential to build a reliable solution. Although we demonstrate our pipeline in a sorghum field, the pipeline can be generalized to other grain species. Our pipeline provides a high-resolution head density map that can be utilized for diagnosis of agronomic variability within a field, in a pipeline built without commercial software.

## Introduction

Plant phenotyping is the foundation of breeding selection process for grain crops and has historically been comprised of a combination of observations by skilled, trained breeders (estimates of crop phenology, disease/pest resistance, visual scores, and counts of heads per planted row) and machine-guided measurements (yield [using a plot harvester], grain size [auto counting and weighing of samples], and grain quality measures with near infrared instruments; for example, [1]). While these standard measures easily capture the yield and grain number per unit area and grain size, they miss the head number per unit area, which is a function of plant sowing density and tillering of plants during growth. Since about 2000, plant phenotyping has started to be augmented using cameras on various ground or aerial vehicles to capture image-based observations of the number of plants in field plots [2], the numbers of grains in panicles [3], and various structural aspects of crops and crop canopies [4–6]. Early detection of head density could replace or augment breeding programs where it is sometimes impractical or inefficient to harvest thousands of plots when the breeder only intends to progress with a small proportion of those plots and may not need the seed at all in the case of a hybrid breeding program. Technologies that allow the estimation of head density also have a practical application in the practice of field scouting to estimate yield during the weeks before the crop matures. Typically, agronomists will make several estimates of head number per unit area (or row) and then, after gauging the size or grain number per head, will multiply these numbers to approximate yield. Hence, accurate estimation of head density using high-throughput image collection becomes of practical use to both plant breeders and field agronomists.

There have been multiple attempts to augment grain yield estimation with machine learning (ML) methods, among which counting/detection methods are predominant in measuring components of Grain yield per unit area = average grain mass * average grain number per head * head number per unit area. These methods can be roughly categorized into regression [7–9], detection [10–12], and segmentation models [13], among which detection models are the most applicable because the bounding-box ground truth is easier to achieve than boundary for segmentation, while providing extra size information that dot-label-based regression fails to provide. Depending on if there is a region of interest proposal step, deep-learning-based detectors can be categorized into 1-stage detectors. Depending on whether there is a region of interest proposal step, deep-learning-based detectors can be categorized into 1-stage detectors [11,14] and 2-stage detectors [12,15]. Generally, 1-stage detectors are believed to be more lightweight and efficient than 2-stage ones, although the latter is considered more accurate. A recent important milestone in real-time 1-stage detectors is You Only Look Once (Yolo) [14], which later was optimized and evolved into several versions, v2 [16], v3 [17], and v4 [18]. All these implementations were based on Darknet [19]. Later, another PyTorch implementation was proposed in [10]. Two-stage detectors have had a longer history. Starting from region-based convolutional neural network (R-CNN) [20], which was proposed to address the problem of selecting the most representative regions by selective search, Fast R-CNN [21] replaces the searching-based region proposals with a convolutional network and then the region proposals are input to a region of interest polling layer to output a fixed size. Faster R-CNN [12] further improves the first-stage frame by using region proposal network, and it became the baseline of later improvements, such as region-based fully convolutional network [22] and mask R-CNN [15].

ML methods have been applied to various grain species. The availability of Global Wheat Head Detection dataset [23,24], composed of 4,700 box-annotated red–green–blue (RGB) images of wheat field images collected over various different locations. The Global Wheat Head Detection attracted the attention of many ML researchers and practitioners in 2 competitions [25,26]. Most methods use existing detection models, such as the Yolo series, Faster R-CNN, and EfficientDet, while Khaki et al. [26] modified Mobilenet [27] detection model to provide a lightweight network. Fourati et al. [25] proposes a Faster R-CNN-based [12] and EfficientDet-based [11] pipeline, which adds a few engineering tricks for the competitions, such as data cleaning, model ensemble, and adding pseudo-labeling to test data. A detailed explanation of engineering tuning methods were discussed by Wu et al. in 2020 [28]. In order to improve the detection performance in different domains, domain adaptation solutions [29,30] have been proposed. Ayalew et al. [29] modified domain-adversarial neural network [31] by combining U-Net [13] with gradient reversal layer [32]. James et al. [30] applied a style transfer method, contrastive unpaired translation [33], on source domain data to make it the same style as the target domain. After using a label cleaning pipeline, the detection model was retrained for adapting to the new target domain. In maize tassel counting, both regression- and detection-based models have been applied, where together with various bounding-box-label-based detection models. Lu et al. propose TasselNet [8] and TasselNetV2 [9], which applied CNN-based regression models to dot-annotated images to count maize tassels; additionally, they released the Maize Tassels Counting dataset, which is composed of 361 dot-annotated maize field images. A detailed comparison between bounding-box-based detection and dot-based regression was discussed in [34].

Sorghum is another grain species that attracts ML methods for aerial RGB images, similar to the previous 2 grain types (wheat and maize). Guo et al. [35] propose a framework to detect sorghum heads, which uses a decision tree-based image segmentation model to binarize the image into sorghum head and nonhead regions, followed by a quadratic support vector machine to classify the regions into heads and nonheads on the basis of geometric features of the segmented regions; the model was evaluated with an F1 score of 0.92 and 0.89 on 2 separate test sets, and the authors made the dataset publicly available which is composed of 1,440 annotated images of sorghum plots. Sarkar et al. [36] successfully trained a RetinaNet [37] model to detect sorghum heads, and the model was evaluated on the dataset collected by Guo et al. [35] with a mean average precision (mAP) of 0.914 at 0.5 IOU (intersection over union). Since sorghum has a relatively simpler head structure when compared with other grain crops such as wheat and maize (a single large head), it requires comparatively less effort to manually label the boundary of the head in images. Therefore, semantic segmentation methods have also been applied for detecting and counting sorghum heads. Lin et al. [38] applied a U-Net [13] model for segmentation of sorghum head regions, followed by contour detection for separating instances of sorghum heads; the model achieved a mean absolute percent error of 0.15 on the test set. Similarly, Malambo et al. [39] trained a 3-class (head, vegetation, and soil) SegNet [40] model to segment RGB images and applied a combination of watershed and connected component detection to separate individual instances; the model achieved 0.94 accuracy for head counting on the test set. Segmentation-based models achieved the best accuracy when compared to regression and detection methods due to the extra dimension that boundary labeling provides. Although sorghum head boundaries are comparatively easier to label, it is still expensive and time-consuming to manually label pixel-level boundaries. To avoid this, Ubbens et al. [41] propose a crop agnostic unsupervised segmentation model for crop organs; the model uses a CNN to recursively label the superpixel segmentation results from simple linear iterative clustering [42] algorithm, and the algorithm achieves an *r*^2^ value of 0.79 for counting sorghum heads on the [35] dataset. Besides sorghum, rice is another important grain species. Because of the irregular shape of the rice grains, most rice yield estimation methods were either based on time-series vegetation index [43] or further explored vegetation index feature points [44]. There are few articles at present working on direct rice grain detection, which were based on segmentation and clustering methods [45,46].

Although these methods provide various models designed for crop panicle detection, which can be used in assisting grain yield estimation. There is no proposal for a standard pipeline that discusses an end-to-end solution, which outlines the procedure starting from data collection and preparation, to model inference and yield specific statistics calculation. In this work, we propose a standardized pipeline to assist deep-learning-based yield and head density estimation for sorghum from RGB images collected via unmanned aerial vehicle (UAV). Our pipeline discusses data collection, data labeling, model training, augmentation techniques, model evaluation, and finally model testing on new field images, and deriving grain yield-related statistics from model inference. We present results from 2 experiments in this work. In the first experiment, we discuss a scenario where a pretrained model trained from a publicly available dataset does not work well for the target domain and provide a guideline for new training data preparation and model evaluation. In the second experiment, we consider a scenario where pretrained models work well for the new target domain, and we demonstrate how to derive head density estimation from the detection results.

The rest of the paper is organized as follows. Materials and Methods introduces the field description, data collection, data preprocessing, data preparation, and the deep learning pipeline. Results of the model evaluation and deployment are described in Results and are discussed and summarized in Discussion.

## Materials and Methods

We aim to provide an end-to-end pipeline for applying a robust detection model on the field images to detect sorghum panicles, so as to estimate the head density across field accurately. To achieve this goal, we could either use publicly available training data or prepare a new dataset from scratch to train a model. In the first experiment, we discuss new dataset preparation. Where RGB images will be collected, preprocessed, and labeled, followed by training and validating the new model. Ground-level images are recommended for training the model because of its high adaptability. In the second experiment, we discuss when a suitable dataset is available and how it will be used in an ML pipeline to run inference on target field images and calculate head density-related statistics to assist yield estimation.

### Experiment 1: Image acquisition and training data preparation

Preparing training data is not a necessary step if suitable public datasets are available. In this experiment, we consider a scenario where training data needs to collected for a target field, and we propose a guideline for data acquisition, model training, and evaluation.

#### Image acquisition - Training data

**Fig. 1.**
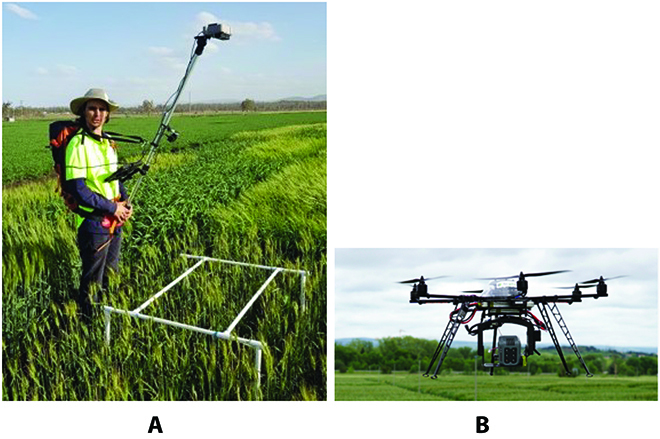
Illustration of 2 image acquisition types. (A) Ground-level acquisition. (B) UAV acquisition.

**Fig. 2.**
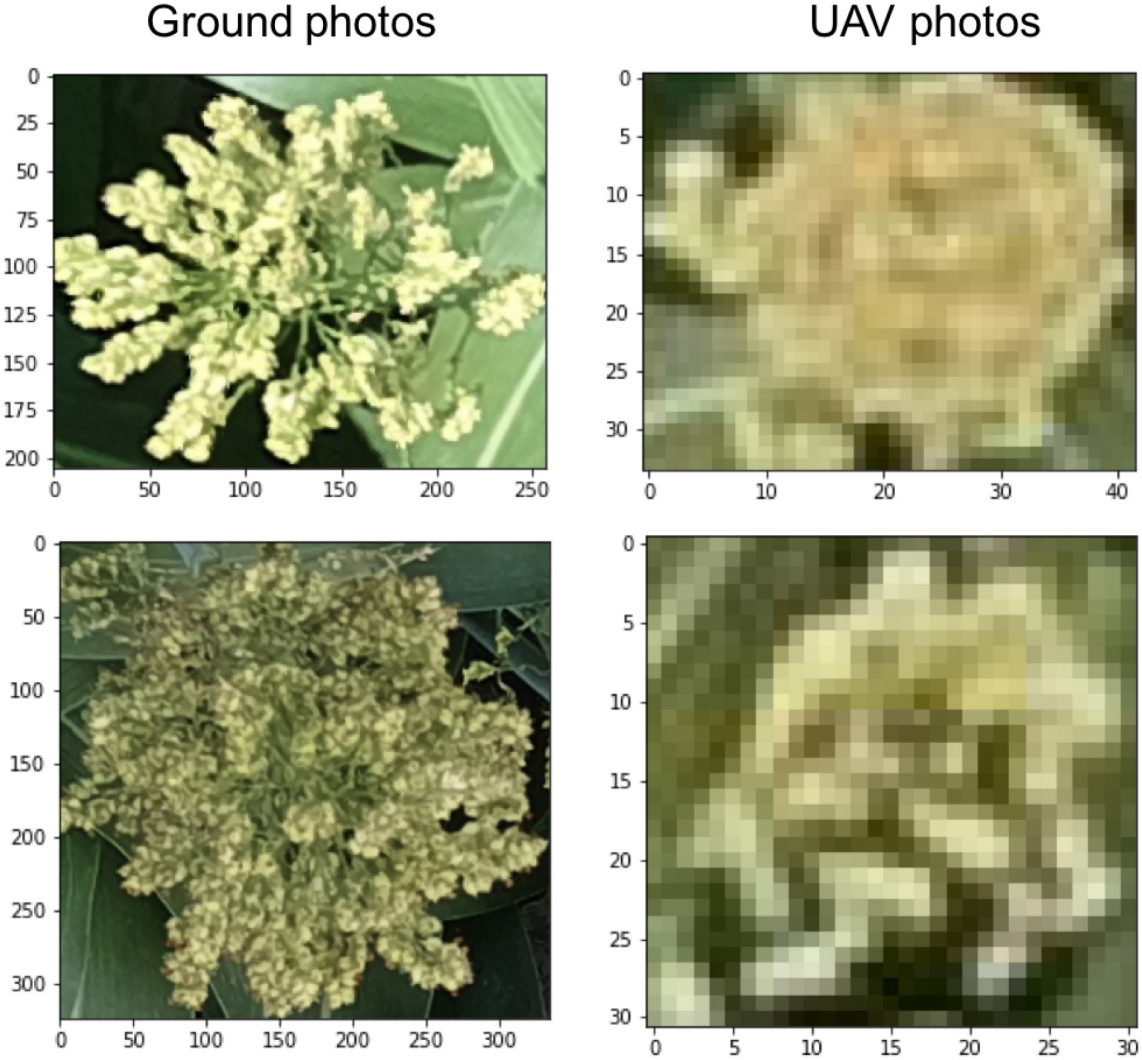
Example comparisons of UAV and ground-level images obtained from the same plot.

There are generally 2 types of RGB image datasets depending on the imaging devices, i.e., ground images and UAV images, as shown in Fig. 1A and B respectively. UAV images are normally captured on higher view (10 to 25 m) than ground images (1.5 to 3 m), thereby having a higher coverage rate and are less time-consuming. However, there is difference between object scales and resolution between 2 imagery types. As shown in Fig. 2, if the grain heads are rescaled into similar scale, UAV images (right-hand side) are blurry compared to ground-level images (left-hand side), where the axes show the original resolution pixels. Analysis of the different impact on detection model for maize was compared in [47]. Similarly, we expect that, for other crop types, due to the loss of texture features, training on high-view blurry UAV images would not achieve as good performance as ground-level images.

**Fig. 3.**
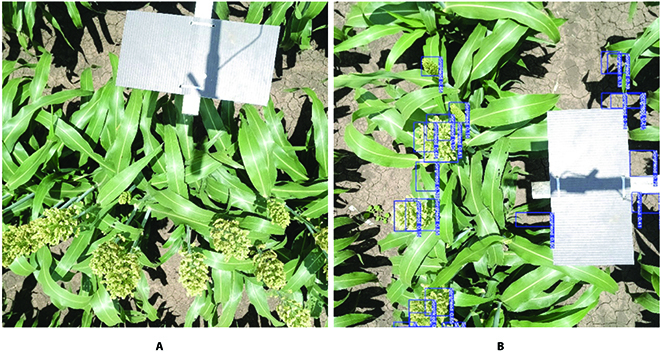
Illustration of sorghum ground images and coarse labels. (A) Illustration of sorghum ground imagery. (B) Coarse sorghum head labels by initial model.

Therefore, we provide an example of ground-level image collection for a sorghum field, and it can be generalized to any crop type. We collected ground level from a breeding trial in December 2021, the crops were in early-mid flowering stage during image acquisition, and the trial was located at the Gatton campus of the University of Queensland (UQ), Australia. The images were collected using an OpenCV OAK-1 camera at ground level. The camera has a 12-MP sensor, and the images were captured at a square 2,160-pixel resolution (4K). The camera was attached to a 1.5-m pole and held on top of the sorghum canopies to capture approximately nadir view images. Additionally, while collecting the images, we placed a reference object in the frame of view. The reference object was used to estimate ground sample distance for the captured images. We used a black cardboard rectangle with a width of 30 cm and a height of 15 cm. Figure 3A shows captured images from sorghum canopies. The pixel count for the size of the reference objects in the images (width of the rectangle) was used to calculate the ground sampling distance (GSD) of the images. The sorghum plants were in the early stages of their development; hence, the color of the heads were green. A total of 165 images of sorghum canopies were collected.

#### Initial model

**Fig. 4.**
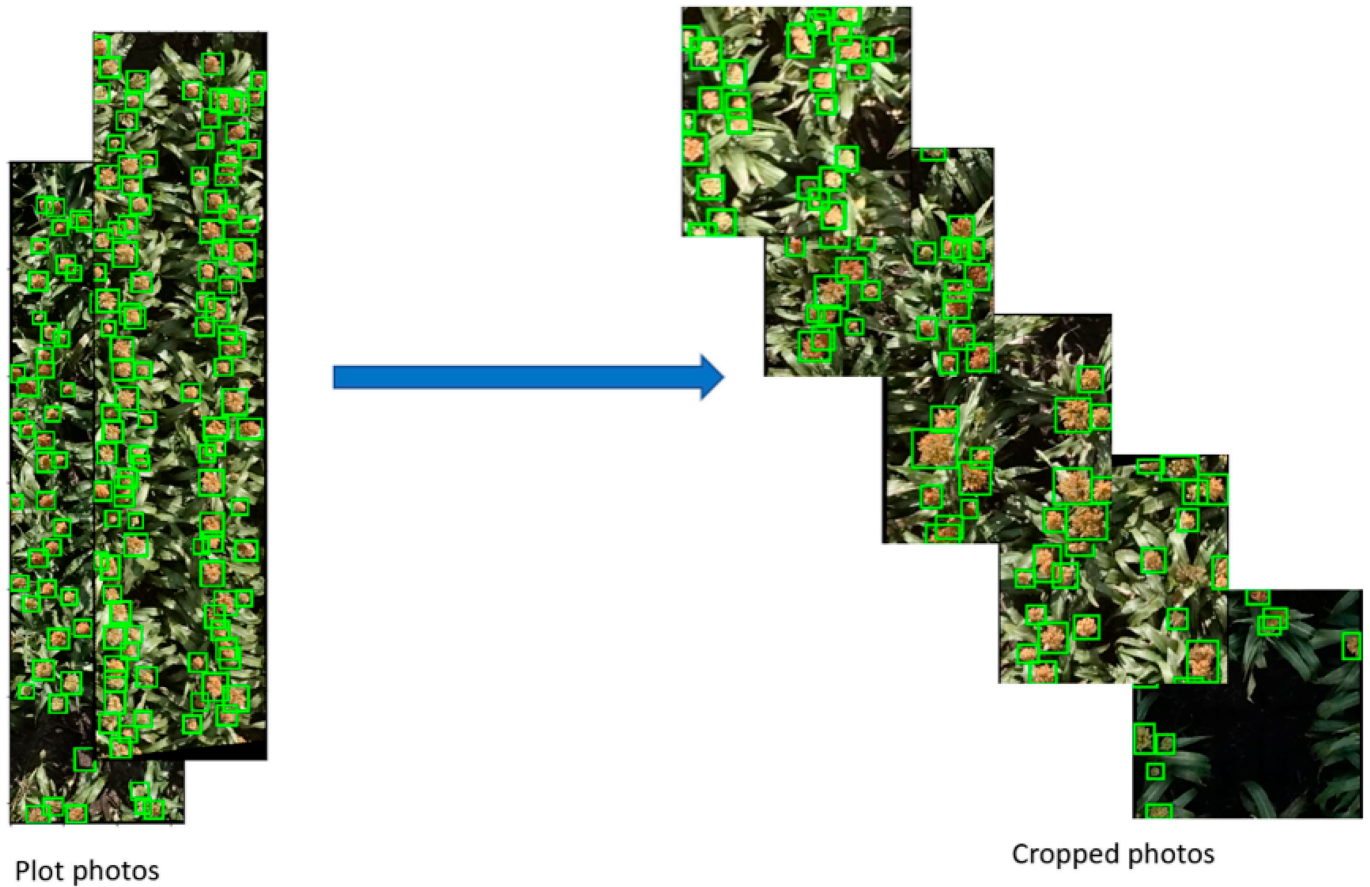
Illustration of the training set for initial model.

The initial model was trained on an open-source sorghum dataset collected by Guo et al. [35] (the dataset is described further in experiment 2, “Detection model training with data augmentation” section). Before training the initial model, the original images in the dataset were cropped into square patches along the length of the plot, using the resolution of the width of the plots. After cropping the images, the size of the dataset was 3,717 images, and there were on average 28 heads per image. These cropped images were used to train a “S” (small) version YoloV5 model [10] (https://github.com/ultralytics/yolov5), with the default yolov5 augmentation pipeline; this would be the initial model. A total of 3,567 images were used as training set and 150 images were used as the validation set. The input resolution for the model was 360 by 360 pixels. The weights for the model were initialized using pretrained MS COCO (Microsoft Common Objects in Context Dataset) [48] weights, and the model was trained for 500 epochs. Figure 4 shows the training set for the initial model. The initial model weights were used to initialize the weights for training the baseline model.

Additionally, another model was trained, the “X” version of YoloV5 using the same dataset, which was used to automatically label the newly collected high-resolution dataset described in the previous section. The next section discusses image preprocessing and annotation steps.

#### Image preprocessing and labeling - Training data

In order to reduce the labeling effort, the images can be partially annotated using pretrained models. If there is no such pretrained model, this step is skipped and all heads need to be manually labeled. In this example, we used the initial model described in the previous section to prelabel the collected ground images. Figure 3B shows the pretrained model predictions for the newly acquired sorghum imagery.

From Fig. 3B, we can see that the sorghum model overestimates the number of heads present in the image. The sorghum model mistakes patches of ground as heads. This is due to the domain shift between the data collected in the field and the dataset used for training the initial model. The initial model was trained on images collected for mid-late flowering stage sorghum canopies via a UAV, while the new data was collected for early flowering stage sorghum canopies via a ground camera. The labels and the images were imported to VGG Image annotator, which is an open-source image annotation tool, and the labels were manually corrected by removing false labels and adding missing ones.

Finally, to obtain a consistent dataset, all images need to be regularized to a similar standard. One of the most important characteristics is object scale, i.e., the size of grain heads in pixels. All images need to be rescaled on the basis of their different GSDs, to keep grain heads in a consistent scale. Therefore, all the images were rescaled to match the GSD (0.25 cm per pixel) of the UAV test set (described in the “Initial model” section). Additionally, in order to reduce the edging distortion that is caused by the camera, all images were center cropped by removing 10% edges.

Figure 7 provides an overview of data preparation, model training, and evaluation.

#### Image acquisition - Testing data

**Fig. 5.**
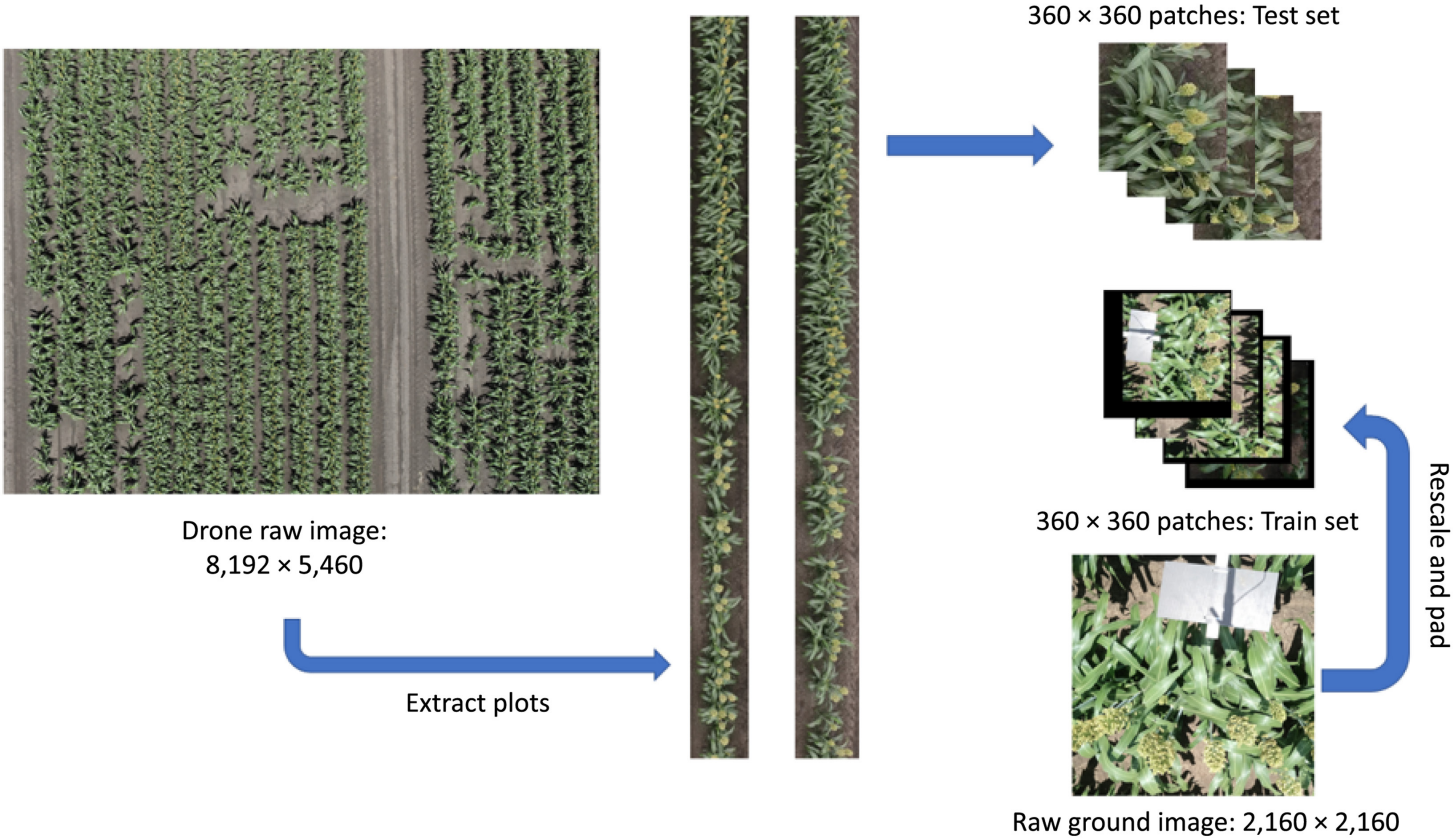
Illustration of the training and test set for different scale images.

**Fig. 6.**
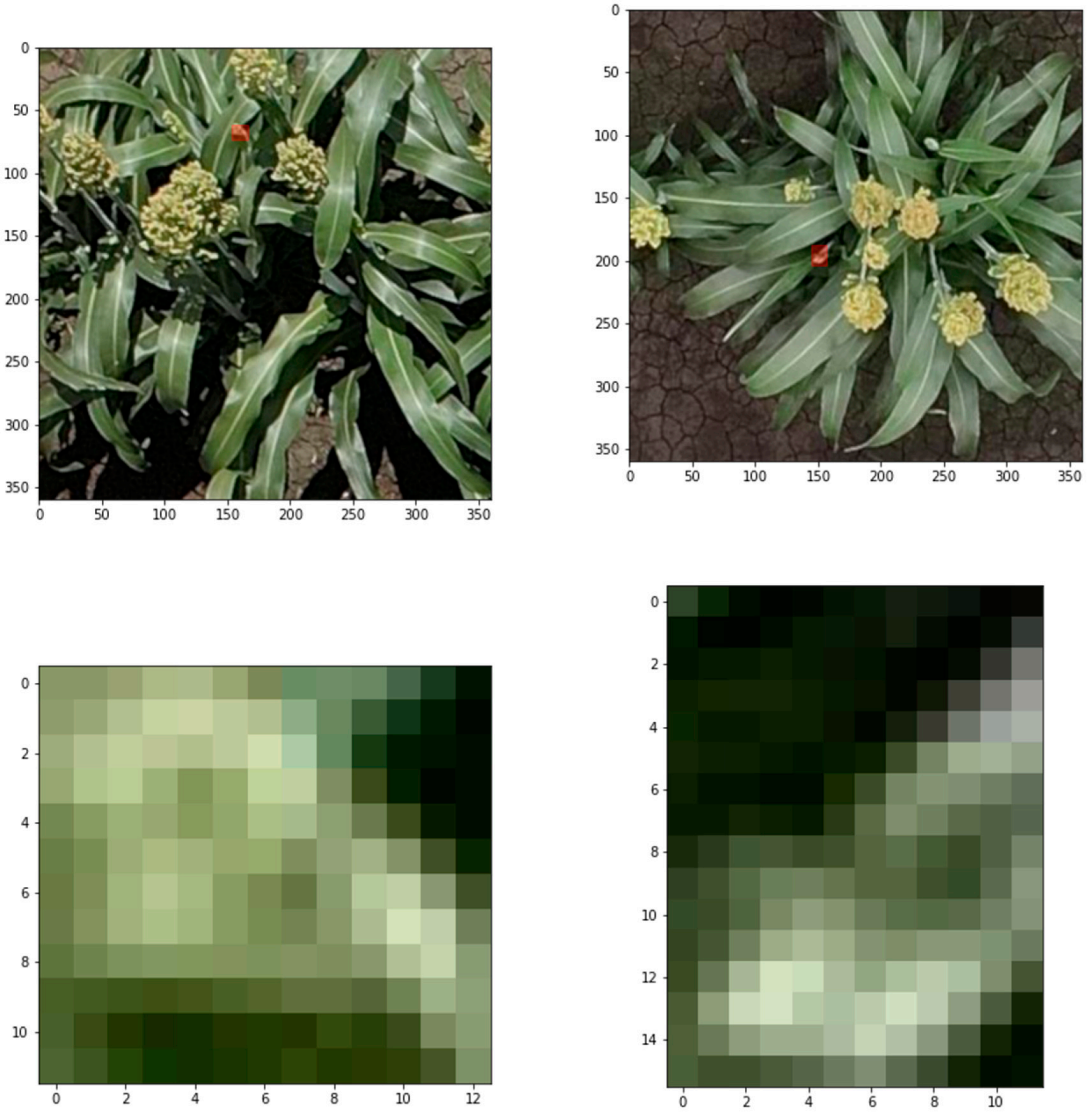
Examples of challenging heads to label in UAV test set.

The test set (UAV images) is composed of sorghum plot photos extracted from the raw imagery captured by the DJI Matrice 300 drone. The drone was flown at 20-m height, and the GSD for the raw imagery was 0.25 cm per pixel. The plots were extracted from the raw imagery and then cropped into 360 by 360 pixel patches. The test set was composed of 97 plot patches that were manually labeled. Figure 5 shows the test set and train set image resolutions. It is worth noting that there is a considerable decrease in the spatial resolution of images when moving from ground to UAV images. As a result, physical presence of some heads in the images needed to be verified in the field, especially for the smaller heads, as shown in Fig. 6. Table 1 provides the raw image acquisition details for the experiment.

**Fig. 7.**
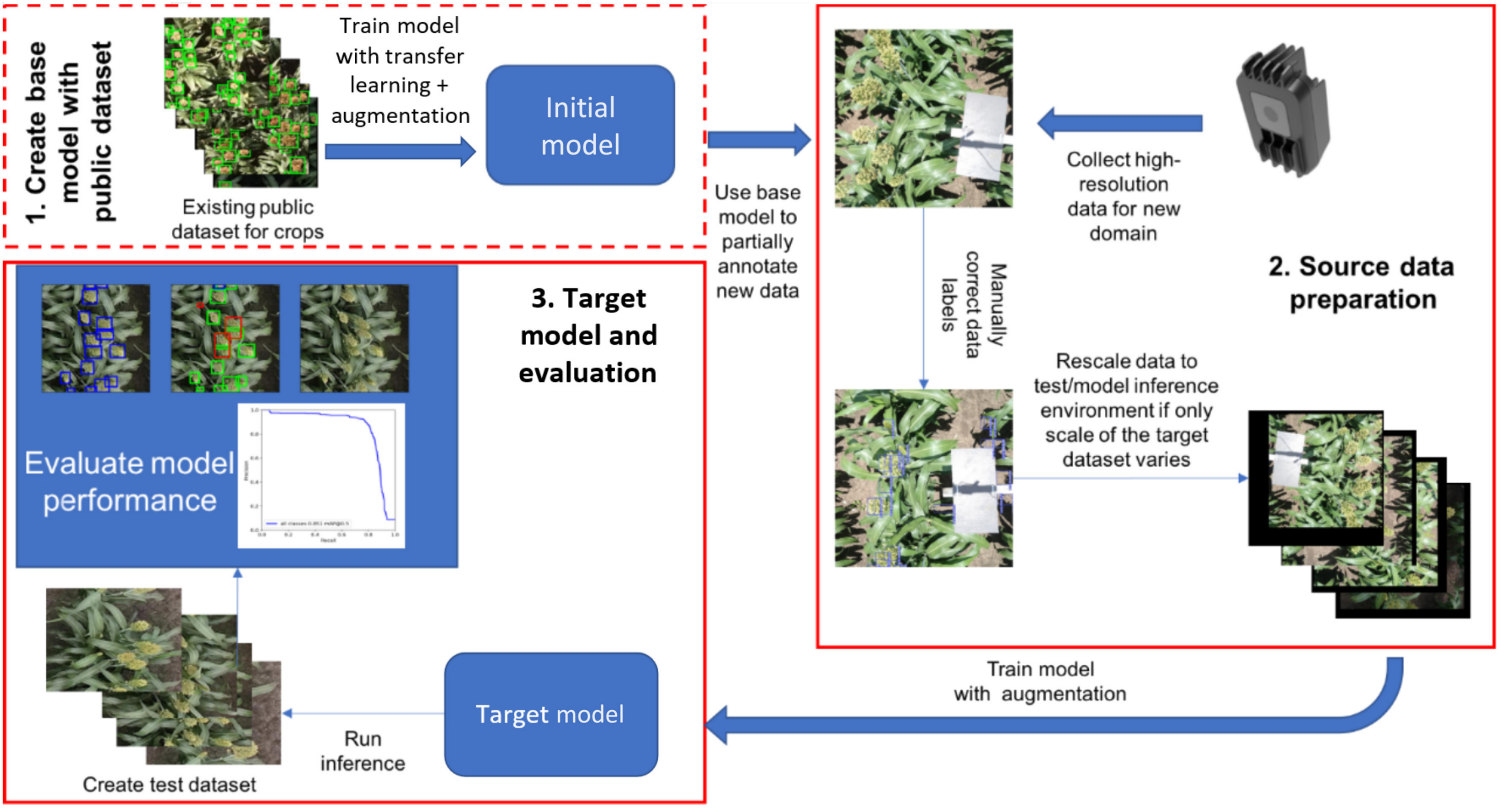
Overview of the source data preparation and model training/evaluation.

**Table 1.** Experiment 1: Train and Inference set image acquisition details

Dataset	Camera	Sensor	Raw Resolution	Altitude	GSD
Train/Valid	OpenCV OAK-1	12 MP,11.04 mm	4,032 × 3,040	1.5 m (approx)	0.028–34 cm/px
Test	DJI Matrice 300	45 MP, 35.9 × 34 mm	8,192 × 5,460	20 m	0.25 cm/px

### Experiment 2: Deep learning pipeline demonstration

Here, we propose a standardized deep-learning-based pipeline for sorghum head detection, to assist grain yield estimation. In this experiment, we consider a different scenario for another field, where a publicly available dataset is suitable for the target domain. We provide an example of training a model on an existing dataset, followed by running inference on new images and calculating relevant statistics.

#### An overview of the pipeline

**Fig. 8.**
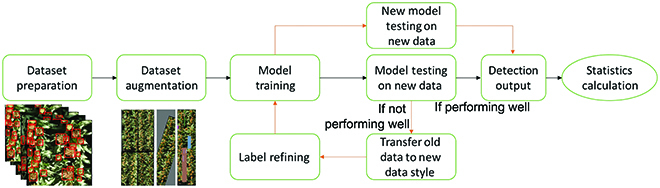
Pipeline of ML-based grain heads detection.

The deep-learning pipeline is as illustrated in Fig. 8, to train a model and test on any new coming fields. In order to maximize the model performance and robustness, augmentation methods would be applied before training (offline augmentation) or during training (online augmentation). The common augmentation methods were reviewed in [49]. After data augmentation for the training sets, we train either a regression model (CSRnet [7], TasselNet [8]), or a detection model (YoloV5 [10], EfficientDet [11], and Faster R-CNN [12]) for head counting. However, even with augmentations, the model performance often deteriorates when working in another dataset (domain) because of domain shifts between 2 datasets. Hence, domain adaptation methods could be applied in specific cases (especially when the target domain is significantly different from the available dataset in terms of image acquisition) to solve this problem [29,30]. Finally, the output is deployed in new field images, and count estimation statics are calculated.

#### Experiment materials

**Training data:** The UQ dataset was collected by the UQ at the QDAF Hermitage Research Station near Warwick, Queensland, Australia (28.21°S, 152.10°E) and was all manually labeled and cross validated by the University of Tokyo [35]. Most of the sorghum plants in the field were in the heading stage at the time of capture. The raw images were collected via a drone flying at 20-m altitude, with a commercial RGB camera with a native resolution of 5,472 × 3,648 pixels, which resulted in an average GSD of 0.45 cm per pixel. The raw images were stitched to construct an orthomosiac image of the field. The dataset is composed of images of pairs of plots extracted from the orthomosaic image, so the final resolution of the plot photos in the dataset is variable because of the plot extraction algorithm. The height of the images approximately ranges between 1,100 and 1,600 pixels and the width of the images ranges between 300 and 500 pixels. There are 1,161 images in total, among which 1,000 images are used for training, while the rest are used for validation.

**Fig. 9.**
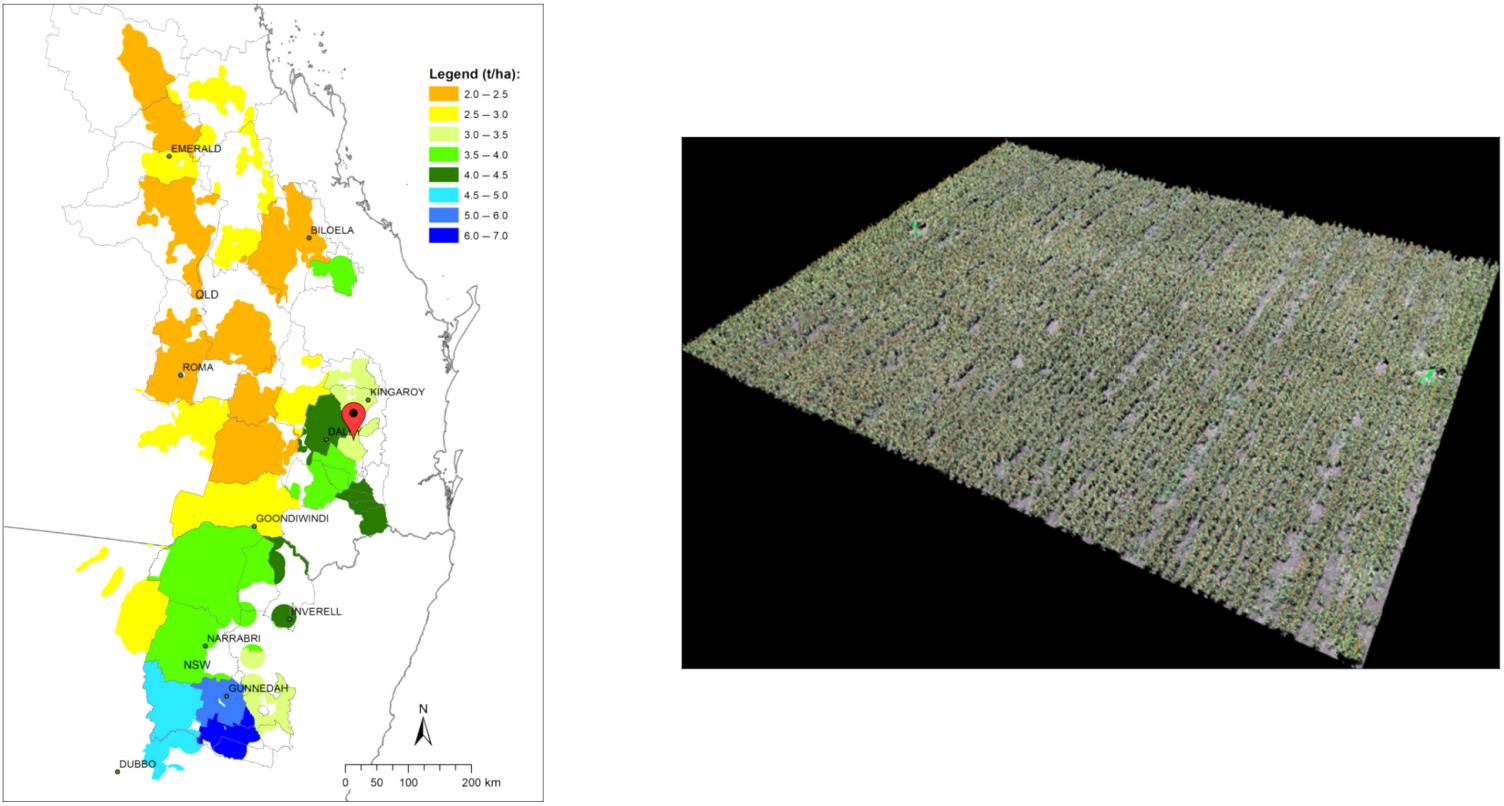
Overview of the study area. (Left) Long-term median yield (2000 to 2020) estimated at shire level for the Australian sorghum production area extending over more than 1,000 km from lower yielding areas in Central Queensland (approximately 23.5°S, shorter-season and lower-water-capacity soils) to higher yielding areas on longer-season, deeper-soil areas in northern New South Wales (NSW) (32.2°S), red marker-approximate trial location [54,55]. (Right) RGB ortho-mosaic imagery of site. QLD, Queensland.

**Testing dataset:** The test dataset was collected from an experimental trial located on an experimental farm at Jondaryan, Australia (27.45°S, 151.53°E), during the late flowering stage in February 2021, as shown in Fig. 9. Sorghum was sown at a planting density of 80 k/ha with a row spacing of 75 cm. The images were captured with DJI Phantom 4 Pro flown at an altitude of 22 m, at a native resolution of 5,472 x 3,648 pixels, resulting in an average GSD of 0.60 cm per pixel. Table 2 provides the image acquisition details for training and testing data.

**Table 2.** Experiment 2: Train and Test set image acquisition details

Dataset	Camera	Sensor	Raw resolution	Altitude	GSD
Train/Valid	Sony DSC-RX100M3	20.1 MP,13.2 × 8.8 mm	5,472 × 3,648	20 M	0.45 cm/px
Inference	DJI Phantom 4 Pro	20 MP,24 mm	5,472 × 3,648	22 M	0.60 cm/px

#### Detection model training with data augmentation

For robust detection performance, the data is normally augmented before training. Here, we recommend using the same augmentation pipeline with YoloV5 [10], so as to imitate real situations in fields. The augmentation pipeline includes mosaic augmentation (imitating various light conditions or cloud occluding sunlight), cutout augmentation (imitating empty growing patches and reducing head density per image), rotating (imitating tilted grain row captures), scaling (different capturing heights and different head sizes), and color augmentation (different lighting conditions and growing stages). After confirming the augmentation pipeline, we can choose either online augmentation or offline augmentation by applying individual or a combination of different augmentation methods to a portion of the training images. Here, we applied online augmentation with the following configuration: every image in a batch will be augmented with a probability of 0.66. Every image selected for augmentation will be first augmented with 1 randomly chosen augmentation out of the aforementioned methods, followed by being augmented by the remaining augmentation methods with an independent probability of 0.2. This configuration allows the model to be trained with a combination of original images, images augmented with multiple combinations.

As discussed earlier, all dot-regression, object detection, and pixel-level semantic segmentation methods can be used in this case. Here, we consider 2 object detection methods, YoloV5 and Faster R-CNN. If the number of the training dataset is small or it is required to train the model quickly, YoloV5 is preferred. On the other hand, if there is sufficient training data and inference time for the model is not a constraint (for example, deploying the model in the field for real time counting), Faster R-CNN can be considered.

**Domain adaptation (optional):** If the new domain is extremely different from the training set, e.g., datasets of 2 different genotypes, the pretrained model may not perform well even after simple image processing techniques. Therefore, domain adaptation can be applied to improve detection performance, so as to facilitate later procedures. This extra domain adaptation method is unsupervised, i.e., no extra labeling is required. Given training dataset as source domain and new field as target domain, source domain data are first transformed into target style with contrastive unpaired translation generative adversarial network [33] that is trained on unlabeled data from both domains, and then the original labels are corrected before being used for training a new detection model that adapts to target domain. In data-label-correction stage, manual cleaning is not necessary, but with a small amount of manual cleaning, the detection performance will be further improved. More details can be found in [30], and it demonstrates a boost of both detection accuracy and robustness with domain adaptation. Because of the similarity between the training dataset and new field data in this experiment, domain adaptation is not necessary in this case.

#### Model inference

**Fig. 10.**
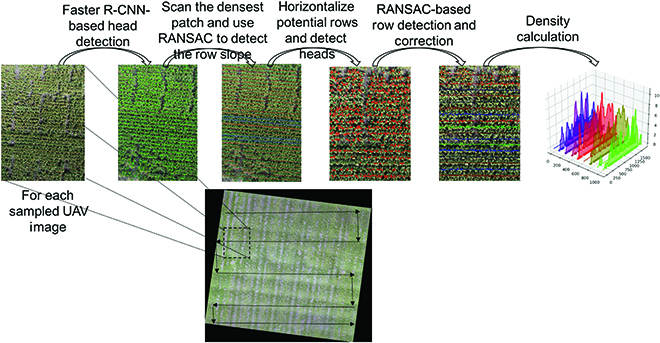
Deployment pipeline.

Here, we provide an example of deploying the pretrained model that is trained on an available sorghum dataset (transfer learning), i.e., UQ dataset [35], on a new sorghum field. The deployment pipeline is shown in Fig. 10. After collecting UAV images in a new field, 10% edges of all images are removed before testing on the pretrained Faster R-CNN. The detection results are used as row detection input. The row detection method that we use here is RANdom SAmple Consensus (RANSAC). Row direction is a key indicator of accurate row detection, especially for scattered images. Therefore, running RANSAC on the densest part per image is performed before on the entire image to locate the dominant direction. Then, all detected rows are compared with the dominant direction, and the image is corrected to be horizontal, to construct the quasi-mosaic field image. Finally, statistics, including image-wise and field-wise row/area density, are calculated on the basis of on each image or entire field.

**Grain head detection:** Grain head detection is the most important step, as it highly influences later procedures. Besides an accurate detection model, it is also important to make test domain images to be similar to the training domain images. Firstly, to remove potential edge distortions, images of new fields are center cropped with removing 10% edges. Then, the image is rescaled to have similar GSD with the training dataset, so that it has similar head density per image, thereby maximizing the detector performance. If the image attributes of training set are different from the test set, i.e., for testing images have higher resolution than training images, Gaussian blurring can be applied to the test set [47].

**Planting row detection:** After achieving all head detection, we use RANSAC [50] on detection results for locating rows. RANSAC randomly select *n* points out of data points to try to find a model that could accommodate most points, where all points that fit the model within the threshold *t* are called inliers. In case that the number of data point are too large and we aim to avoid iterating all data points, it is often set a maximum number *k* of iterations that is allowed in the algorithm and a number *d* of close data points that are required to assert that a model fits well to data. In our work, we use the center point of each head detection bounding box as the data point and iteratively use the generic RANSAC algorithm for each row until it finds all row models that fit all data points. Row models are a series of lines, and each line *i* is represented by:

$$y_{i}=a_{i}\cdot x_{i}+b_{i}$$ (1)

where *a_i_* is the slope and *b_i_* is the line intersection with the left edge of the image. The pseudo-code is described in Algorithm 1.

**Quasi-mosaic image construction:** “Quasi-mosaic” is the term used in this paper to refer to a composite image of the entire field, constructed by roughly stitching the raw UAV images together. For UAV images, 2 consecutive images normally overlap, and the percentage of overlaps greatly depend on the UAV flying speed. In order to better visualize the entire field and demonstrate the statistics across the entire field, the quasi-mosaic image is constructed by combining all field images and removing the overlaps. This method is much faster and less intensive compared to applying methods based on feature-matching algorithms to stitch images together.

Firstly, a grid of size *m_x_* × *m_y_*, where *m_x_* and *m_y_* are smaller than the image coverage respectively, is built for the entire field. Given that the Global Positioning System (GPS) location of each grid center (*i*,*j*) is *L*_(*i*,*j*)_, the location of each grid is within *L*_(*i*,*j*)_ ± *m_x_*/2 from west to east and *L*_(*i*,*j*)_ ± *m_y_*/2 from north to south. Therefore, the image that is closest to the grid center is selected to fill in the grid location and construct the quasi-mosaic field image. All the images are reoriented, by rotating them along the planting row direction (calculated in the previous step). Provided that the GPS location of each image selection *L*_*i*,*j*_ and GSD *G*, field coverage of each image is known, the rotated images are cropped and plugged into their respective grid locations. Alternatively, if a more visually accurate and consistent representation of the field is required, mature structure-from-motion-based photogrammetry software like Pix4D or Agisoft may be considered.

**Statistics calculation:** After the previous procedures, statistics for each image and for the entire field can be calculated to better explain the sorghum yield estimation. For each image, headcounts *C_r_* along the row and headcounts across the image *C_i_* are calculated for understanding the head density, while gaps that are greater than 50 cm counted across the images to account for significant gaps. Provided that all planting rows are horizontal in the image, and GSD *G* of image of size *W* × *H* is known.

$$D_{r}=\frac{C_{r}}{W\cdot G},$$ (2)

and head density across image

$$D_{i}=\frac{C_{i}}{W\cdot H\cdot G^{2}}.$$ (3)

For the entire field, we can visualize the panicle density by showing the moving average / moving sum of the head count per meter/square meter.

**Algorithm 1** RowDetection

1:  **function** RowDetection(*data, model, n, k, t, d*)

2:    *allBestFits* = []

3:    **while** point numbers of *data > n* **do**

4:      *iterations* = 0

5:      *bestF it* = *Null*

6:      *bestErr* = 9999999

7:      **while** *iterations < K* **do**

8:        *maybeInliers* := *n* randomly selected values from data

9:        *maybeModel* := *model* parameters fitted to *maybeInliers*

10:        *alsoInliers* := Ø

11:        **for** every *point* ∈ *data* & ∈*/ maybeInliers* **do**

12:          **if** *point* fits *maybeModel* with an *error < t* **then**

13:            add *point* to *alsoInliers*

14:        **if** the number of elements in *alsoInliers > d* **then**

15:          *betterModel* := *model* parameters fitted to all points in *maybeInliers* & *alsoInliers*

16:          *thisErr* := a measure of how well *betterModel* fits these points

17:          **if** *thisErr < bestErr* **then**

18:            *bestF it* := *betterModel*

19:            *bestErr* := *thisErr iterations*+ = 1

20:      append *bestF it* to *allBestFits*

21      remove all *points* ∈ (*maybeInliers* & *alsoInliers*) in data

22:      *iterations = 0*

23:      *bestF it = Null*

24:      *bestErr* = 9999999

25:    **return** *allBestFits*

## Results

### Experiment 1: Model training and evaluation for new dataset

In this section, we train the model on the ground imagery that we collected and evaluate the model performance on UAV images. At the site, 164 ground images were collected to create the training set for the baseline model. Of these, 154 images were used for training, and 10 validation images were selected to sample the diversity of head size, color, and structure and were manually labeled (i.e., edited after partial automatic labeling) as described in the materials and m ethods section “Image preprocessing and labeling - Training data”. We rescaled the images in the new training dataset (ground images) to match the resolution of the test set. The test set (UAV images) is composed of sorghum plot photos extracted from the raw imagery captured by the DJI Matrice 300 drone, with a GSD of 0.25 cm per pixel. A total of 97 plot patches were extracted and manually labeled, as described in the materials and methods section “Image acquisition - Testing data”.

Figure 12A shows the performance on the validation set (ground images), and Fig. 12B shows the performance of the model on the test set (UAV images). The mAP for model detections falls from 0.958 to 0.851 when going from ground to UAV images. Upon manual inspection of the results, it was found that for UAV images the model missed small heads that were in the very early stages of development; furthermore, in some instances, the model also wrongly detected the midrib of the leaves and patches of soil as heads. These issues may be primarily attributed to the altitude of the drone during image capture. The spatial resolution of the drone images is lacking detail for the model to accurately detect and classify very small heads in some instances; even during the manual labeling of these images, it was challenging to ensure the veracity of labels as discussed in the “Initial model” section.

**Fig. 11.**
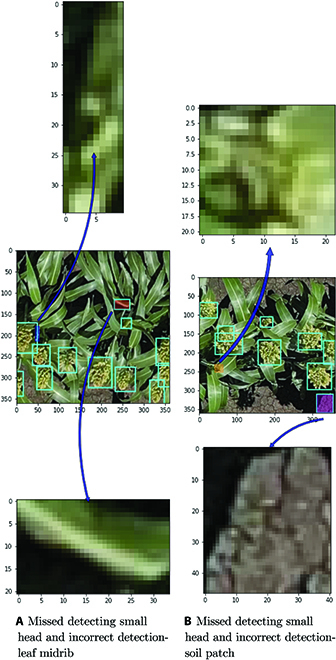
Missed heads and wrong detections by model. Top: Zoomed view of missed head. Middle: Original image with detection boxes, wrong detection (magenta patch), missed detection (orange patch). Bottom: Zoomed-in wrong detection.

**Fig. 12.**
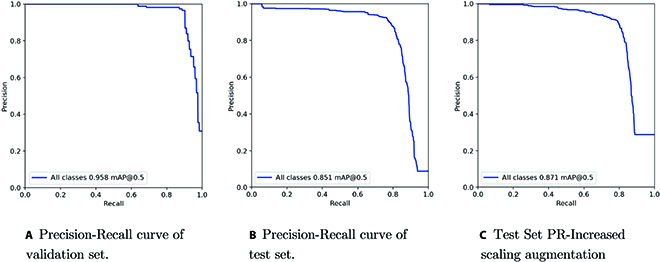
Detection model performance. Left: Precision-recall curve for detection model on validation set. Middle: Precision-recall curve for detection model on test set. Right: Precision-recall curve for detection model (retrained with increased scale augmentation) on test set.

To attempt to remedy the scale issue, the model was trained with a stronger image scale augmentation configuration of −50% to +50% image rescaling, as opposed to the initial configuration of −10% to +10% image rescaling. This led to a slight increase in model performance, the mAP increased from 0.851 to 0.871, as shown in Fig. 12C. However, the model still continued to struggle with missing and wrongly detecting small heads. The examples demonstrated in Fig. 11 shows the heads missed and wrongly detected by the model.

### Experiment 2: Deep learning pipeline - Inference and statistics

**Fig. 13.**
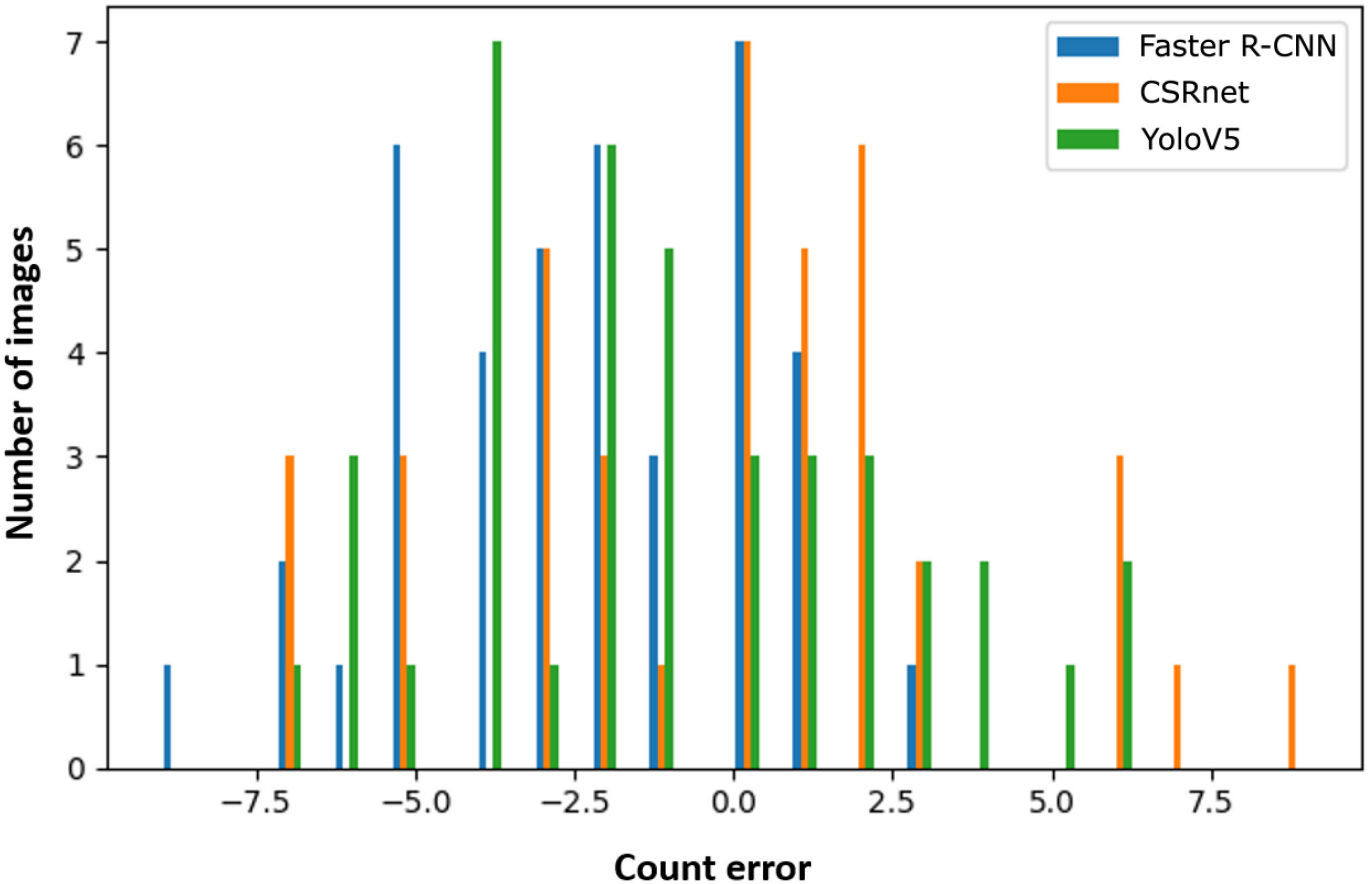
Error histogram of various models.

Various models are trained and tested on the available sorghum dataset [35], as shown in Table 3. As there are only bounding-box labels for the UQ dataset, the segmentation model is not included in the comparison and 1 representative model of each category is compared, i.e., regression models: CSRnet, 1-stage detectors: YoloV5, and 2-stage detections: Faster R-CNN. According to the performance on the validation set, Faster R-CNN is selected to test on the new sorghum field dataset. It is observed that regression models have better accuracy than detectors. The error histograms of models are shown in Fig. 13. Both detection models are prone to under detect, while the regression model (CSRnet) is more balanced. Also, it is worth noting that if the detection models are to be deployed in a real time counting application, like counting from live video feeds, 1-stage detector models should be preferred over 2-stage detectors as they have fewer parameters to train, and faster inference time, Table 3 also compares the total number of trainable parameters for all models.

**Table 3.** Head count performance of various models (104 average heads per image)

Category	Model	MAE	RMSE	# of million params
Regression model	CSRnet	2.74	3.23	16
1-stage detector	YoloV5-L	2.95	3.50	47
2-stage detector	Faster R-CNN-ResNet 101	2.87	3.64	60

MAE, mean absolute error; RMSE, root mean square error

**Fig. 14.**
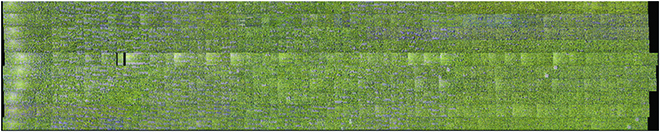
Quasi-mosaic image for the entire field.

**Fig. 15.**
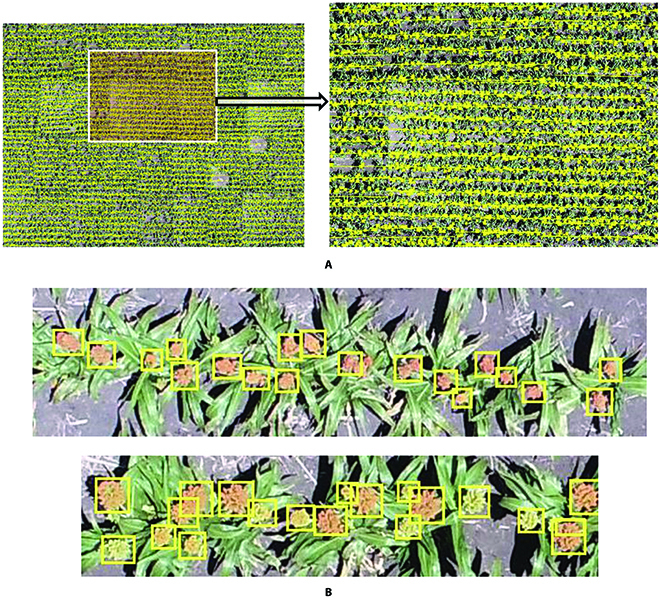
Row detection showing in quasi-mosaic image. (A) Row detection showing in quasi-mosaic image. (B) Zoomed-in detections.

In order to calculate the field-wise statistics, quasi-mosaic is constructed according to GPS locations of each image according to the quasi-mosaic construction method in the “Model inference” section. The quasi-mosaic image for this sorghum field is shown in Fig. 14. It is good enough to depict the entire field and show the head count information across the entire field. In order to show the details of the detection and row connections between 2 grids, a zoom-in area is shown in Fig. 15. It shows that after row detection and rotation, rows of each grid connect well with their neighbor grid, so it is effective to use GPS information of UAV to construct the quasi-mosaic images with grids. Figure 15B shows the zoomed in head detections. Furthermore, we evaluated the accuracy of the model detections based on a small test, composed of 50 plots sampled from the raw UAV images that were manually annotated. The model had an over all accuracy of 93.59% on the test set.

**Fig. 16.**
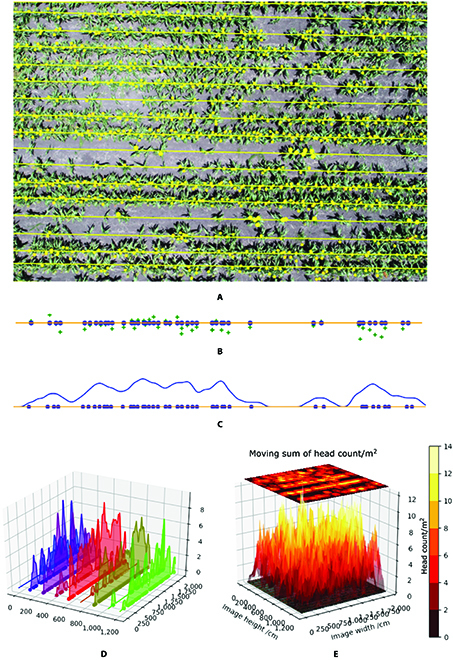
Image-wise head density visualization. (A) Head detection and row detection. (B) Dot regression on nearest row. (C) One-dimensional moving sum head count and Gaussian smoothing for each row. (D) 3D display of head density per row. (E) Head density across entire image.

On the basis of the trained Faster R-CNN model, sorghum heads are detected in all new field images and are predicted as bounding boxes. The centers of the predictions are provided to RANSAC model for row detection, as shown in Fig. 16A, and later statistics calculations are based on these detection results. Image-wise headcount calculation for Fig. 16A is illustrated in Fig. 16B and C, and visualization of head density across row / area is shown in Fig. 16D and E. The GSD of the field images is 0.53 cm/pixel, so we rescale the image, so that each pixel of the image is 1 cm. The unit of the density is head counts/m and counts/m^2^ respectively for Fig. 16D and E.

**Fig. 17.**
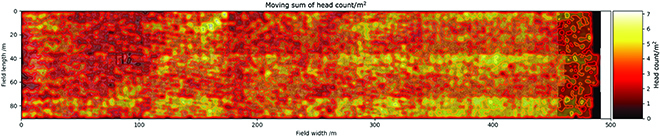
Density map across entire field.

Figure 17 displays the density map of heads in the field. An area of 100 × 100 pixels represents 1 m × 1 m in field. Therefore, heads are counted continuously within the area of 100 × 100 across the quasi-mosaic image, and the counts move 1 step from left to right and up to bottom to calculate headcounts across the entire field. In order to smooth the final results, a two-dimensional Gaussian filter with a size of 100 × 100 is applied on the counts. The filter sliding window convolution multiplies with 100 × 100 head counts of the quasi-mosaic image. The sum of the Gaussian filter is 1 in a 1 m × 1 m scale and convolution, so that it only smoothes the visualization and does not influence the physical meaning of the headcount density. Therefore, the unit of density is head counts/m^2^, and head counts vary from 0 to 6.68/m^2^.

## Discussion

In this work, we introduce a comprehensive pipeline to apply deep-learning detection models for sorghum head counting, to assist grain yield estimation via RGB UAV images. Our work is complementary to existing literature for crop head detection. We take various real-world variables into consideration, including data preparation, model validation, inference, and deriving yield-specific metrics. We aimed to outline a practical and end to end pipeline from prototype to inference, for sorghum head detection.

For training a suitable model for your target field, you can either collect your own dataset or you can start with a suitable public dataset. We consider both these scenarios in the 2 experiments presented in our paper.

In our first experiment, we propose a pipeline for dataset preparation and model evaluation for a new target field. We collect a dataset for a field with sorghum plants in the early stage of heading. Models trained on publicly available sorghum datasets, which focus on mid to late flowering sorghum plants, were not suitable for our target field. Therefore, we collected a new ground image dataset, followed by using a combination of semiautomatic and manual labeling to annotate the dataset. Finally, we adapt our ground image dataset to train a model to detect heads from UAV images. We train and validate our model only on ground images, and we evaluate our model on a separate test composed only of UAV images, in order to observe the generalization capability of the model. We found that the mAP of the model dropped from 0.958 (when evaluated on ground images) to 0.871 when evaluated on UAV images. The model performance dropped because of poor detection performance for small heads in the early stages of development. Indicating the spatial resolution of the (for this specific experiment) UAV images may not be optimal for the detection of early-stage small heads. One apparent solution is to increase the spatial resolution of the images to improve the detection performance. Alternatively, for future work, we suggest including deep-learning-based super resolution methods to improve spatial resolution [51].

In our second experiment, we provide a pipeline describing the deployment of deep-learning-based detection models on another sorghum field. The overview of the pipeline is as follows: (a) Multiple deep learning methods are considered and compared. If only accurate head counts are needed, regression models could provide more accurate results than detectors. However, if the field needs to be analyzed with more details, detector models are necessary. (b) We propose a RANSAC model for row detection, which uses the head detection results identify planting rows, in order to analyse gaps between rows and variation in head density. (c) Heading density /m^2^ head counts are visualized on a per-image basis and across field.

Although our work tries to provide a comprehensive head number estimation pipeline from collecting data to final analysis visualization, it might still encounter additional problems. For instance, 1 limitation is that the RANSAC-based row detection method is fully contingent on the head detection results, which enlarges the error for row detection results. Later, it could be replaced with deep models of semantic row detection based on the raw images directly. In addition, research on semantic row detection will be conducted. Finally, it is also worth mentioning that the detection models, image preprocessing, and data augmentation techniques discussed in this paper are focused on popular CNN-based architectures. As research progresses in the field of computer vision and object detection, and newer frameworks and architectures are introduced, e.g., transformer architecture-based models like the Swin tranformer [52], image preprocessing and augmentation methods must be carefully reconsidered and examined in light of newer model architectures [53], before incorporating newer state of the art models into the pipeline. For our future work, we intend to test our pipeline on tasks of more grain types, e.g., wheat and maize yield estimation.

## Data Availability

The datasets and models used in the findings of this study are available on request from the corresponding author. They are not publicly available as all data collected in the study is part of the GRDC project “UOQ2002-08RTX”.
